# Low-intensity pulsed ultrasound (LIPUS) prevents periprosthetic inflammatory loosening through FBXL2-TRAF6 ubiquitination pathway

**DOI:** 10.1038/srep45779

**Published:** 2017-04-05

**Authors:** Xiang Zhao, Gangsheng Zhao, Zhongli Shi, Chenhe Zhou, Yunlin Chen, Bin Hu, Shigui Yan

**Affiliations:** 1 Department of orthopaedic surgery, the second affiliated hospital, School of Medicine, Zhejiang University, Hangzhou, China; 2 Institute of orthopaedic research, Zhejiang University, Hangzhou, China; 3 Department of Orthopaedic Surgery, Yiwu Central Hospital, the affiliated hospital of Wenzhou Medical College, Yiwu, China; 4 Department of Orthopaedic Surgery, Ningbo sixth hospital, China

## Abstract

Previous studies have shown that Low intensity pulsed ultrasound(LIPUS) prevents polyethylene-debris-induced periprosthetic loosening *in vivo*, but the details of the mechanism by which it does so remain unclear. In this article, we used polyethylene debris induced RAW 264.7 cells as the *in vitro* model, and tested the effect of LIPUS on this model. Changes in the level of inflammatory cytokines, cell proliferation, and apoptosis were assessed. Gene overexpression and siRNA technique were applied, and the levels of expression of FBXL2, TRAF6, ERK, and related inflammatory cytokines were also measured. Results indicated that FBXL2-mediated TRAF6 ubiquitination and degradation also plays an important role in aseptic periprosthetic loosening process, and LIPUS prevents such loosening by strengthening this pathway.

Joint replacement can dramatically reduce joint pain, restore limb function, and increase quality of life. It is one of the most effective methods of addressing end-stage joint arthritis. However, the long-term revision rate has also puzzled doctors and patients^1^. The rate of aseptic loosening rate has been reported to be as high as 75%, and most of the patients have to undergo revision within 10–15 years of the initial surgery^2^.

The inflammatory microenvironment is the main pathophysiological cause of aseptic loosening^3^. Polyethylene debris induces activation of osteoblasts, causing periprosthetic osteolysis which leads to joint loosening. Though many methods have been proposed to prevent such inflammation, including improving friction interface to reduce debris and drugs to inhibit inflammation, the former is questioned for its high cost and high complication rate, and the latter is reported to have poor outcomes. Finding an effective, noninvasive and well-tolerated technique to prevent aseptic loosening has become very important.

Tumor necrosis factor receptor-associated factor (TRAF) plays an important role in the process of inflammation. There are a total of 7 factors in the TRAF family^4^. They share a highly conserved carboxyl-terminal domain. This domain mediates interaction with transmembrane tumor necrosis factor receptors (TNFR), mediates downstream signals such as NF-κb, and influences cytokine expression, leading to severe inflammatory swelling, organ failure, and shock. Some studies have confirmed that TRAF6 plays a key role in the differentiation of macrophages^5^,^6^. In the process of aseptic loosening, the polyethylene debris from the prosthesis mediates the inflammatory microenvironment with the following steps: First, the endocytosis of these particles by tissue cells induces the release of inflammatory chemokines; secondly, monocyte-macrophage gather following these chemokines; third, these monocyte-macrophages can be activated by polyethylene debris or by the secretion of inflammatory cytokines such as IL-1β and IL-33. On the cell membrane, IL-1βacts on TLR2/4 by binding with IL-1βR, influences the function of TRAF6 by Myd88, and finally activates the NF-κB signal pathway, leading to osteolysis.

TRAF can be degraded by FBXL2 through ubiquitination^5^,^6^,^7^. It is reported that FBXL2 binds with c-terminal of TRAF, mediates its degradation by ubiquitination^8^,^9^,^10^,^11^,^12^, and prevents inflammation in murine lung injury model infected with *Pseudomonas aeruginosa* PA103^13^. Inhibition of FBXL2 or knock out of its gene aggravates such inflammation. As an aseptic inflammation, periprosthetic loosening has an inflammatory cascade effect similar to that of bacterial infection, and monocyte-macrophage cells play a key role in both processes. It is here speculated that FBXL2-mediated TRAF6 ubiquitination and degradation are also the key processes in aseptic periprosthetic loosening.

FBXL2 may be a suitable target for low-intensity pulsed ultrasound (LIPUS). LIPUS can subject tissue cells to shear stress by cavitation mechanical effects, mediate intracellular molecular signaling, and the extracellular environment, promote its proliferation and differentiation, and induce extracellular matrix synthesis^14^. Some studies have shown that LIPUS also takes effect by influencing the binding of the ligand and receptor^15^. Our previous studies have shown that ultrasound can reduce inflammatory swelling in rat hip infection model^16^. LIPUS also can induce osteogenesis and inhibit polyethylene particles induced in periprosthetic osteolysis in the current rabbit model^17^. However, the details of mechanism are still not clear. The following hypothesis is here proposed: in the aseptic inflammatory microenvironment induced by debris, FBXL2 can mediate ubiquitination and degradation of TRAF6. LIPUS can also induce the expression of FBXL2, inhibit the downstream inflammatory signal pathway, reduce the inflammatory cascade effect, and keep the stability of microenvironment.

## Results

### MTT test for cell proliferation

Neither polyethylene debris nor LIPUS were found to have any obvious influence in RAW 264.7 cell proliferation (Table 1).

### Flow cytometry for cell apoptosis

**Figure 1:**
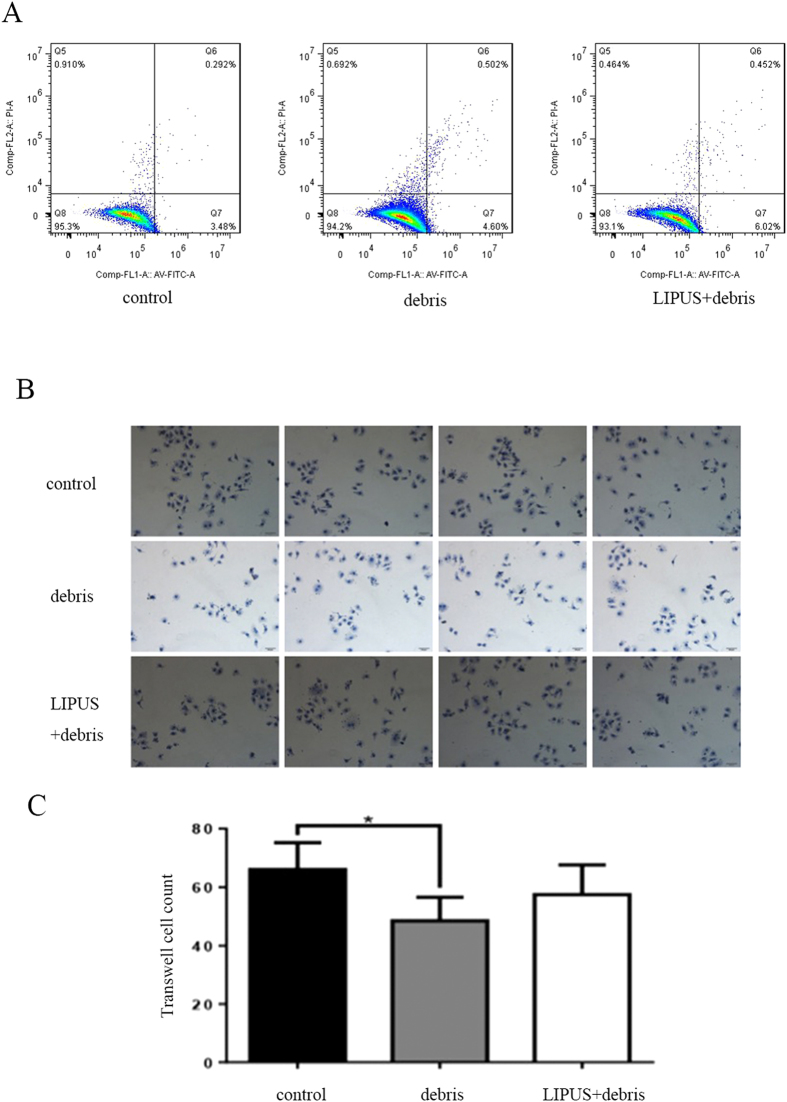
Part (**A**): the result of cytometry. Part (**B** and **C**): the migration of RAW 264.7 cells is dramatically reduced in debris group, and LIPUS can prevent such reduction(*p < 0.05 vs. control group).

No obvious influence of polyethylene debris or LIPUS was found on apoptosis. However, LIPUS tended to be associated with a greater rate of apoptosis than in the debris group (Fig. 1A, Table 2).

### Transwell test for cell activity

The polyethylene debris group was associated with far less rates of cell migration than the control group (*P* < 0.05), and LIPUS was found to prevent this difference from emerging (*P* > 0.05) (Fig. 1B–C).

### Inflammatory cytokine testing

**Figure 2:**
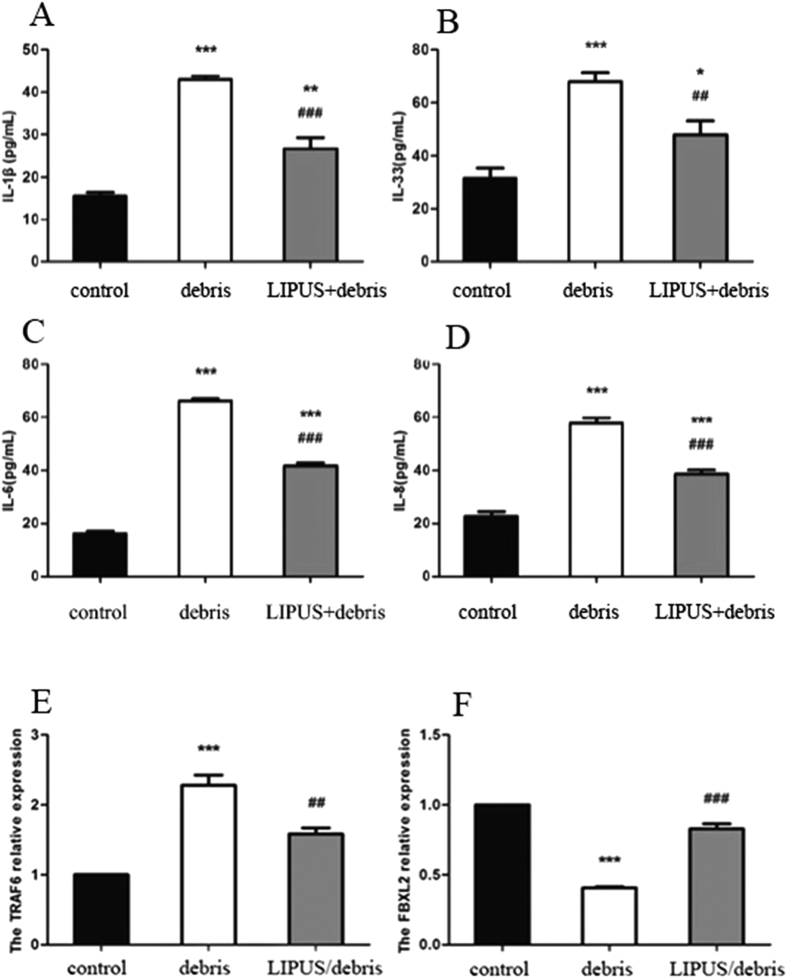
Part (**A,B,C** and **D**): the change of cytokine protein expression. In debris group, all inflammatory cytokines(IL-1β, IL-33, IL-6 and IL-8) increase, whereas LIPUS inhibits these cytokines. Part E and F: the change of FBXL2 and TRAF6 expression. Debris induces TRAF6 and inhibits FBXL2; while LIPUS inhibits TRAF6 and induces FBXL2(*p < 0.05, **p < 0.01, ***p < 0.001 vs. control group; ^##^p < 0.01, ^###^p < 0.001 vs. debris group).

Polyethylene debris dramatically increased the expression of IL-1β, IL-33, IL-6, and IL-8. LIPUS was found to reduce the rate of such expression and prevent inflammation (Fig. 2A–D).

### Protein signal test

#### qRT-PCT

There was far less FBXL2 expression in the polyethylene debris group than in the control group (*P* < 0.001), and there was more expression of TRAF6 (*P* < 0001). The LIPUS group showed more FBXL2 expression and less the TRAF6 expression than the debris group. Results indicated that LIPUS can influence the stability of TRAF through FBXL2 (Fig. 2E and F).

### Western blot

**Figure 3:**
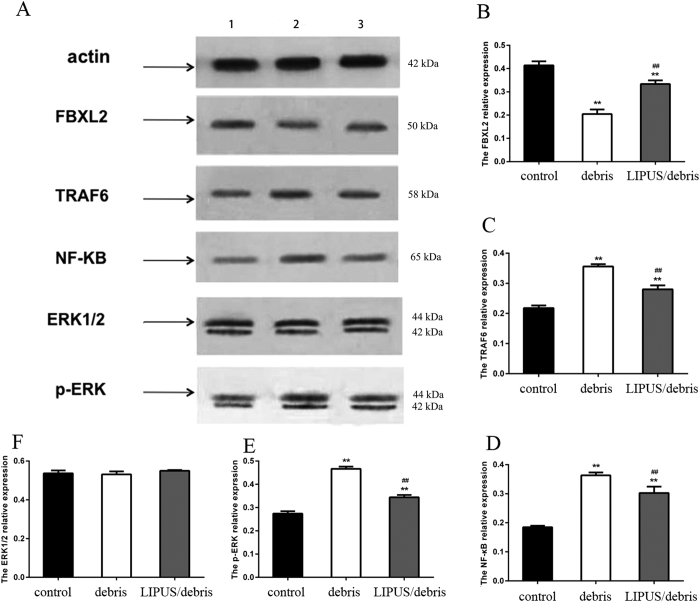
Group 1 is control group; group 2 is debris group; and group 3 is LIPUS+debris group. This figure shows the change of proteins expressions(FBXL2, FBXL6, NF-**κ**B, and ERK) in western blot(**p < 0.01 vs. control gourp; ^##^p < 0.01 vs. debris group). Full-length blots are included in the Supplementary Figure S1.

The polyethylene debris group showed less expression of FBXL2 and more expression of TRAF6, NF-κB, and p-ERK than the control group. LIPUS was found to increase the expression of FBXL2 and decrease the expression of TRAF6, NF-κB, and p-ERK (Fig. 3).

### Co-immunoprecipitation (Co-IP)

**Figure 4:**
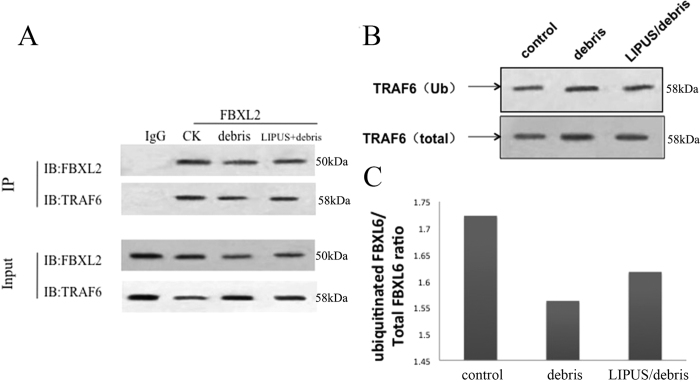
Part A shows the result of CO-PI, indication the interaction between FBXL2 and TRAF 6. Part (**B** and **C**) show the ratio of ubiquitinated TRAF6/original total TRAF6. Comparing with debris group, LIPUS can higher the ratio of ubiquitinated TRAF6. Full-length blots are included in the Supplementary Figure S1.

This test confirmed that FBXL2 and TRAF6 had interactions in every group (Fig. 4A).

#### Ubiquitination

The debris group showed a lower percentage of ubiquitinated TRAF6 (ub-TRAF6/total TRAF6) than the control group, and LIPUS reversed this difference. (Fig. 4B and C).

### Overexpression and interference of FBXL2

#### Gene transfection and siRNA

**Figure 5:**
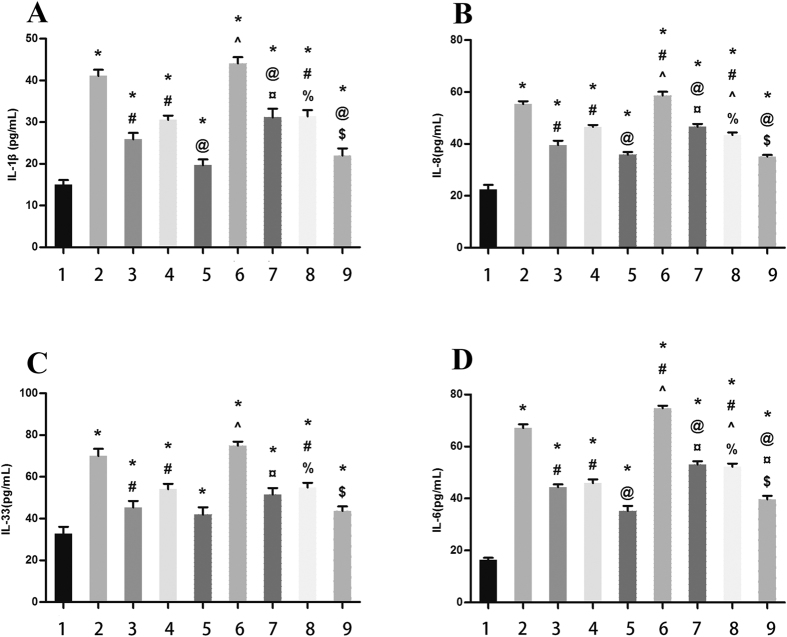
The level of inflammatory cytokines(IL-1β, IL-33, IL-6 and IL-8). Group 1: control; group 2: debris; group 3: LIPUS/debris; group 4: FBXL2 overexpression+debris; group 5: FBXL2 overexpression+LIPUS/debris; group 6: FBXL2-siRNA+debris; group 7: FBXL2-siRNA+LIPUS/debris; group 8: ERK 1/2-siRNA+debris; group 9: ERK1/2-siRNA+LIPUS/debris. *Means p < 0.05 vs. Group 1; ^#^means p < 0.05 vs. group 2; ^@^means p < 0.05 vs. Group 3; ^means p < 0.05 vs. Group 4; ^¤^means p < 0.05 vs. Group 5; ^%^means p < 0.05 vs. Gourp 6; ^$^means p < 0.05 vs. Group 7.

The levels of IL-1β, IL-33, IL-6, and IL-8 were all increased in the debris group (Group 2) than control group (Group 1). LIPUS was found to inhibit such inflammation and decrease the levels of these cytokines (Group 3). In the group with FBXL2 overexpression and debris (Group 4), levels of IL-1β, IL-33, IL-6, and IL-8 were lower than in the debris group (Group 2). LIPUS was found to further decrease these inflammatory cytokines’ level (Group 5). If FBXL2 was silenced, debris dramatically increased IL-1β, IL-33, IL-6 and IL-8 level (Group 6), and LIPUS prevented such increase (Group 7). If ERK1/2 were silenced, the levels of inflammatory cytokines were also lower (Group 8 and Group 9) (Fig. 5).

**Figure 6:**
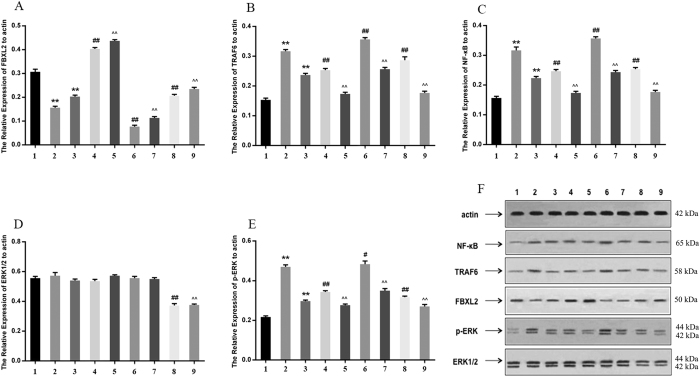
The expression of FBXL2, TRAF6, NF-κB, ERK1/2 and p-ERK. Group 1: control; group 2: debris; group 3: LIPUS/debris; group 4: FBXL2 overexpression+debris; group 5: FBXL2 overexpression+LIPUS/debris; group 6: FBXL2-siRNA+debris; group 7: FBXL2-siRNA+LIPUS/debris; group 8: ERK 1/2-siRNA+debris; group 9: ERK1/2-siRNA+LIPUS/debris. **p < 0.01 vs. control group; ^#^p < 0.05, ^##^p < 0.01 vs. debris group; ^^p < 0.01 vs. LIPUS/debris group. Full-length blots are included in the Supplementary Figure S1.

Debris could higher the level of TRAF6 and NF-κB, while lower the level of FBXL2 (Group 2). LIPUS had the opposite effect (Group 3). When FBXL2 was overexpressed, the levels of TRAF6 and NF-κB were decreased, and LIPUS was found to enhance this effect. Silencing of FBXL2 increased the level of TRAF6 and NF-κB, and silencing of ERK1/2 decreased the level of TRAF6 and NF-κB (Fig. 6).

## Discussion

This study demonstrated that TRAF6 plays an important role in the process of aseptic inflammatory loosening caused by periprosthetic debris, and LIPUS was found to inhibit such inflammation through increasing the level of FBXL2, and further increasing ubiquitination of TRAF6.

In the current study, polyethylene debris was used to create an *in vitro* aseptic loosening model. The metal to polyethylene interface is currently the most popular choice in joint replacement. Periprosthetic loosening caused by polyethylene debris is still the gold standard of joint loosening^17^,^18^,^19^. This could help us best simulate microenvironmental changes in RAW 264.7 cells.

The current study confirmed the hypothesis that the TRAF6 also plays an important role in the process of aseptic periprosthetic loosening, as it does in the septic inflammatory process. Previous studies have indicated TRAF6 took critical part in the processes of sepsis-induced cardiac dysfunction(Ma *et al*.)^20^, ischemia-reperfusion injury(Liu *et al*.)^21^, immunity response to virus infection(Wei *et al*.)^22^, and innate immunity(Panda *et al*.)^23^. In the current study, there were far higher levels of TRAF6, IL-1β, IL-6, IL-8, and IL-33 in the debris group, demonstrating TRAF6 plays a key role in the process of aseptic inflammatory loosening.

TRAF6 can be degraded by ubiquitination^24^. Ubiquitin is one kind of heat shock protein, with molecular weight between 0.5 and 1.0 kDa. Ubiquitin exists in most of cells. It can mark and degrade proteins. This ubiquitination process includes many enzymes, such as E1 ubiquitin-activating enzyme, E2-conjugating enzyme and E3-ubiquitin ligase. The Skp-cullin1-F box (SCF) superfamily contains one kind of E3 ligase. Each is composed of at least 4 polypeptides, including Skp1, Cullin/CDC53, F-Box, and Rbx1. Among them, F-Box directly connects to phosphospecific domain, and is the key factor in the process of recognition of and binding to targeted proteins by E3 ligase. Fbxl2 is one member of the F-Box protein family. It contains a F-box functional domain and a CAAX sequence. Fbxl2 can mark different proteins, mediate their degradation, and finally inhibit their proliferation and induce apoptosis. However, little is known about the role of FBXL2 in periprosthetic inflammatory microenvironment. Chen *et al*. of the University of Pittsburgh discovered that FBXL2 can mediate degradation of TRAF in alveolar epithelial cells and finally inhibit inflammation induced by *Pseudomonas aeruginosa*^25^. FBXL2 binds with c-terminal of TRAF, mediates its degradation by ubiquitination, and prevents inflammation; inhibition of FBXL2 or knockout of its gene will aggravate such inflammation. As an aseptic inflammation, periprosthetic loosening has similar inflammatory cascade effect as bacterial infection, and monocyte-macrophage cells play key roles in both processes. In the current study, the level of FBXL2 decreased and the TRAF6 increased after addition of debris. This indicated that the debris expressed FBXL2 and promoted the expression of TRAF6. In the latter part of the current study, TRAF6 levels were found to be dramatically decreased after overexpression of FBXL2, indicating the ubiquitination of TRAF6 by FBXL2 plays a key important role in the process of aseptic loosening process.

Previous animal studies have shown that LIPUS has anti-loosening effect in periprosthetic inflammatory loosening process, but the details underlying the mechanisms remain unknown^17^. In recent studies, it has been hypothesized that LIPUS exerts its effects in cells through mechanical stresses^14^. Nakao *et al*. from Japan applied LIPUS on osteoblasts. They found that LIPUS inhibited TLR4-MyD88 complex formation, and further reduced inflammatory response induced by lipopolysaccharide through inhibition of TLR4 signal transduction^15^. However, lipopolysaccharide is the main component of gram-negative bacteria compartments. The inflammation induced by lipopolysaccharides is much more like septic inflammation, which is different from the aseptic loosening inflammation process induced by polyethylene debris. However, little is known about the role of osteoclast by LIPUS in such processes. Previous studies showed different intensities of LIPUS had different effects on osteoclast. Monici *et al*. and Chen *et al*. using comparatively higher intensity(100 mW/cm^2^ and 125 mW/cm^2^, respectively) found decreased osteoclast actively^26^,^27^, whereas Feres *et al*. using lower intensity(30 mW/cm^2^) found increased resorption activity by osteoclast^28^. In the current study, RAW 264.7 cells served as the test subjects, and results showed the levels of IL-1β, IL-6, IL-8, and IL-33 to be reduced, confirming the anti-inflammatory effect of LIPUS(200 mW/cm^2^) in a polytherine-debris-induced aseptic loosening model. Further, results also showed increased levels of FBXL2 and decreased levels of TRAF6 after application of LIPUS. This effect was strengthened by overexpression of FBXL2 and weakened by siRNA interference of FBXL2, indicating that the strengthened ubiquitination of TRAF6 induced by FBXL2 plays a key role in the anti-inflammatory process caused by LIPUS in periprosthetic loosening model. The increased percentage of ubiquitination of TRAF6 in LIPUS group also confirmed this. However, the exact mechanism by which LIPUS increases the level of FBXL2 is still not clear. Some studies have shown that such as ERK1/2 in the cell membrane can receive mechanical signals and mediate the expression of different genes and proteins^14^. ERK1/2 MAPK pathway is complex. It is the downstream signal in the inflammatory process, but it also includes several negative feedback loops, such as those of the dual-specificity phosphatase (DUSP) family^29^,^30^. It is here reported that DUSP genes can be induced by ERK1/2 MAPK pathway activation, and these DUSPs in turn dramatically inhibit the expression of ERK1/2^31^,^32^,^33^. The current study showed ERK1/2 expression to be increased in the polyethylene group, indicating that the ERK1/2 signal plays a role in aseptic periprosthetic loosening. Results also showed that ERK1/2 levels were lower in the presence of LIPUS. The negative feedback regulation of ERK1/2 may explain the decreased ERK1/2 in LIPUS+debris groups. Further study in this field is needed.

The results of the current study have shown that neither debris nor LIPUS has any statistically significant influence on cell apoptosis in RAW 264.7 cells. However, the LIPUS group tended to have a higher rate of apoptosis. Suzuki *et al*. used LIPUS on zebrafish scales (containing both osteoblasts and osteoclasts) for 20 min. Results showed that LIPUS can cause osteoclast apoptosis in their model^34^. The current study is different from Suzuki’s study. First, the model used in that work was a co-culture system (osteoblast and osteoclast), while the one used in the current work contained only pure RAW 264.7 cells. Osteoblasts have a big influence on osteoclast apoptosis. Second, debris were used to create periprosthetic loosening inflammatory environment, this environment may influence the effect of LIPUS on RAW 264.7 cells. These two reasons account for the vague differences among our groups.

The current study showed the transwell rate of RAW 264.7 cells to be dramatically reduced by polyethylene debris, and LIPUS was found to prevent this reduction. Mediero A *et al*. found netrin-1 to be highly expressed in a debris-induced rat osteolysis model. Netrin-1 is a neuroimmune guidance cue. It can inhibit the migration of macrophages, which means that it is an inflammatory factor^35^,^36^. This can explain the reduced transwell rate of RAW 264.7 cell by polyethylene debris in our study. However, the expression of netrin-1 is induced by section of inflammatory cytokines. LIPUS was found to prevent the section of these cytokines and inhibit expression of netrin-1, preventing its inhibition on migration of macrophages.

The current study has several limitations. First, it was performed *in vitro*. Second, the exact mechanism by which LIPUS increases the level of FBXL2 is still not clear. Further studies are needed in the future.

## Conclusion

FBXL2-mediated ubiquitination and degradation of TRAF6 plays an important role in the process of aseptic inflammatory periprosthetic loosening. LIPUS can induce the expression of FBXL2, inhibit the function of TRAF6, and finally prevent aseptic inflammatory periprosthetic loosening. More studies should be performed to establish the details of the mechanism by which LIPUS affects osteoclast cells.

## Methods

This *in vitro* study was approved by the Ethics Committee of Second Affiliated Hospital, School of Medicine, Zhejiang University, China. All experiments were performed in accordance with guidelines for the use of cell lines in biomedical research. No experiments on animal or human were applied in this study.

RAW 264.7 cell lines were obtained from ATCC. MTT was purchased from Sigma. Annexin V-FITC/PI test box was from Bebo Bio. The transwell was from BD Biosciences (353097). IL-33, IL-6, and IL-8 ELISA kits were purchased from NeoBioscience. SYBRGreen PCR and reverse transcription test boxes were from Thermo. FBXL2 antibody (K-12) and TRAF6 antibody (H-274) were from Santa Cruz. NF-κB antibody and ERK1/2 antibody were from Proteintech. Ub antibody (P4) was from Santa Cruz and MG132 was from Sigma.

An LIPUS (Next Sound^TM^ Ultrasound Healing System, YZB/Zhe0550-2003; Nexus Biomedical Devices, Inc., Santa Clara, CA, U.S.) was used to treat RAW 264.7 cells in accordance with the manufacturer’s instructions. The average intensity was 200 mW/cm^2^, the pulsed frequency was 1.5 MHz, the repetition rate was 250 Hz, and the pulse lasted 0.2 ms^16^. The LIPUS generating system with six individual ultrasound transducers was previously described^37^,^38^. The culture plate was placed above these transducers with a thin layer of coupling gel^39^,^40^. The wells vertically above the transducers were used for experiments.

The transwell test was repeated four times, and other tests were all repeated three times in every group.

### Cell culture

RAW 264.7 cells were cultured in Ham’s F12K Medium (Hyclone) supplemented with 10% fetal bovine serum (Hyclone) at 37 °C in a humidified atmosphere of 5% CO_2_ in air. The samples were divided into three groups: 1) control group; 2) polyethylene debris group, in which polyethylene debris was added to the medium during culture; 3) LIPUS group, in which the polytheylene particle was added to the medium, and the cell were treated by LIPUS for 20 min.

For transfection and siRNA, the cells were divided into 9 grours:1) control group; 2) debris group; 3) LIPUS+debris group; 4) FBXL2 overexpression+debris group; 5) FBXL2 overexpression+debris+LIPUS group; 6) FBXL2-siRNA+debris group; 7) FBXL2-siRNA+debris+LIPUS group; 8) ERK1/2-siRNA group; 9) ERK1/2-siRNA+debris+LIPUS group

### MTT testing

Cells (5 × 10^4^ cells/ml) were seeded into 96-well plate with 100 μl in each well (Bogoo Biotechnology Company, Shanghai, China) for 24 h in the dark. After receiving treatment in different groups, the cell survival rate was determined by an MTT assay. 10 μl MTT was added to in each hole and cultured for 4 h. After removing the liquid, 200 μl DMSO was added and the dish was shaken for 10 min. Then the OD value was tested. This assay was performed as a regular procedure and the absorbance at 492 nm was recorded using a microplate reader (BIOTEK ELx800, BioTek, San Diego, CA, U.S.) against the reference value at 690 nm.

### Cell cycle

After treatment in each group, cells were seeded in six-well plates with 3 ml medium for 24 h. Then cells were trypsinized and centrifuged at 1000 r/min for 3 min. The supernatant was discarded, and the cultures were washed with 2 ml PBS at pH 7.2. They were resuspended in cold 70% ethanol for fixation and stored at −20 °C for 4 h. At the time of analysis, cells were centrifuged at 1000 r/min for 3 min, resuspended in 1 ml PBS, and filtered through at 55 μm nylon mesh to remove big clusters. Then 50 μg/ml of propidiumiodide (PI) (Sigma Aldrich, St. Louis, MO, U.S.) and 100 μg/ml of RNAse were added (Sigma Aldrich, St. Louis, MO, U.S.) to stain nuclear DNA and remove RNA from the samples, respectively. Samples were incubated for 30 min in the dark. Then 200 μl sample was tested by flow cytometry, and data were analyzed using CELL Quest software.

### Cell apoptosis assays

Cells (5 × 10^4^ cells/ml) were seeded six-well plate with 3 ml in each well (Bogoo Biotechnology Company, Shanghai, China) for 24 h in the dark. Then, 24 h after treatment in different groups, cells were harvested after trypsinization and centrifugation at 1500 r/min for 5 min. Cells were washed with PBS and resuspended in 300 μl binding buffer, to which 5 μl Annexin V-FITC was added. After 15 min of incubation, 10 μl was added to the mixture, followed by 10 min of incubation in the dark. The quantity of apoptotic cells was determined by flow cytometry (BD Biosciences).

### Transwell testing

After treatment, cells in all three groups were starved for 24 h in serum-free medium and then digested. The cells were washed twice with PBS and re-suspended in serum-free Opti-MEM medium. Transwell assays were adopted. Three chambers were set in each group, and each chamber held 200 μl cell suspensions (2 × 10^5^ cell/ml). A total of 800 μl 10% FBSF12K medium was added to the lower chamber, and then the cells were incubated at 37 °C in 5% CO_2_. Migration test: after 24 h, the chamber was washed with PBS and fixed with 4% paraformaldehyde for 30 s, and then dyed with 5% crystal violet for 20 min.

### Cytokine testing

IL-1β, IL-33, IL-6, and IL-8 levels were measured with specific enzyme-linked immunosorbent assay kits, in accordance with the manufacturer’s instructions. Samples were attached to a microporous plate containing antibodies to each cytokine. The sample diluent and antibodies marked with horseradish peroxidase were added. The absorbance was measured at 450 nm, and the concentration was calculated by a standard curve.

### qRT-PCR

Total RNA was extracted from cells using Trizol reagent (Sigma-Aldrich) according to the manufacturer’s instructions. cDNA was prepared using a Revert-AidTM First Strand cDNA Synthesis Kit (Fermentas, Burlington, ON, Canada) in accordance with the manufacturer’s protocol. To perform real-time PCR, each 20 μl RT-PCR mix contained 10 μl of SYBR Premix Ex Tag, 1 μl of PCR forward Primer, 1 μl PCR Reverse Primer, 1 μl cDNA and distilled water and 7 μl ddH2O. qRT-PCR was performed on an ABI 7500 real-time PCR machine. Conditions were as follows: hold stage was 95 °C for 30 s, cycling was 40 cycles of 95 °C for 5 s and 60 °C for 34 s. The dissolution profile was 95 °C for 15 s, 60 °C for 1 min, and 95 °C for 15 s. The primer sequences of FBXL2 and TRAF6 genes are listed in appendix.

### Western blot analysis

Levels of FBXL2, TRAF6, NF-κ, ERK1/2, and p-ERK were measured using Western blot analysis. SDS-PAGE and immunoblotting were conducted according to set standard procedures. After treatment in each group, the medium as removed and precooling lysate was added (containing 0.25% sodium deoxycholate, 1% Triton X-100, 1% Nonidet P-40, 4 mM EDTA, 10 μg/ml leupeptin, 10 μg/ml aprotinin, and 1 mM phenylmethylsulfonyl fluoride) on ice. Then the cells were harvested into clean EP tube and centrifuged at 12,000 rpm for 20 min, and 1.5 ml supernatant was collected. In this 1.5 ml supernatant, 1.5 μl was collected for protein concentration testing, and the other was added to the addition of 0.4 ml loading buffer (containing 10% SDS, 5% β-mercaptoethanol, 15% glycerol, 0.01% bromophenol blue, and 200 mMTris-HCl; pH 6.7) in boiling water for 5 min and stored at −80 °C.

The protein concentration was tested using a Protein Assay Kit (Beyotime Institute of Biotechnology, Shanghai, China). Then a 0.5 mg/ml standard protein at concentrations of 0, 1, 2, 4, 8, 12, and 16 μl was added to the 96-well plate as a reference. Then 1 μl of each test sample was also added also. In every well, lysate was added to bring the total volume to 20 μl. Then 200 μl BCA was added, and the samples were kept at 37 °C for 30 min. The absorbance was recorded at 492 nm, and the concentration was calculated using the standard curve.

For the SDS-Page electrophoresis, the proteins were transferred onto nitrocellulose filter membranes. Membranes were incubated at room temperature for 2 h and probed overnight at 4 °C with anti- FBXL2, anti- TRAF6, anti- NF-κB, anti- ERK1/2, anti- p-ERK, and anti-actin. Membranes were probed with a second antibody for 1 h and visualized with enhanced chemiluminescence. The figure was screened, and software Image J was used for analysis.

#### Co-IP

FBXL2 antibody and protein A/G beads were added to 1 mg RAW 264.7 cell lysates. After 6 h, wash buffer was used to wash the samples 5 times, and anti-FBXL2 and anti-TRAF6 were used to test CO-IP.

### Cell transfections

Total RNA was isolated and reverse transcription was performed followed by quantitative real-time PCR with SYBR Green qRCR mixture as described. PCR-based approaches were used to clone FBXL-2 into pcDNA3.1D/v5-his (Invitrogen) for constitutive expression in cells. All mutant constructs were generated using PCR-based methods with appropriate primers. RAW 264.7 cells were plated in 48 well plates at the density of 2.8 × 10^5^ cells per well, and the cells were cultured for 18 h. Then, 12 h after transfection, cells were moved to six-well plates, delivered into different groups, and incubated for further 18 h.

### siRNA treatment

The primer sequences of FBXL2, ERK1 and ERK2 genes are listed in appendix. RAW 264.7 cells were plated in 48-well plates at a density of 2.8 × 10^5^ cells per well, and the cells were cultured for 18 h. The growth medium was removed, and AccellsiRNA delivery media containing 1 μM Accell Non-Targeting Pool siRNA (control) or AccellSMARTpool Mouse FBXL2 siRNA (Thermo Fisher Scientific) was added to the cells. Then, 12 h later, the cells were moved to six-well plates, delivered into different groups and incubated for a further 18 h.

### Image Processing and Statistical analysis

Image J 4.7(National Institutes of Health, Bethesda, Maryland, USA) was used to make the Gray-scale analysis. Using SPSS19.0 software, mean ± SD was used for inflammatory cytokine and transwell cell count. Single factor analysis of variance(one-way ANOVA) was used for three or more groups comparasion. P < 0.05 was considered statistically significant.

## Additional Information

**How to cite this article**: Zhao, X. *et al*. Low-intensity pulsed ultrasound (LIPUS) prevents periprosthetic inflammatory loosening through FBXL2-TRAF6 ubiquitination pathway. *Sci. Rep.* **7**, 45779; doi: 10.1038/srep45779 (2017).

**Publisher's note:** Springer Nature remains neutral with regard to jurisdictional claims in published maps and institutional affiliations.

## Supplementary Material

Supplementary Information

## Figures and Tables

**Table 1:** One result of MTT.

Group	OD value		
control	1.101	1.065	1.048
debris	1.056	1.036	1.047
LIPUS+debris	1.038	1.045	0.993

**Table 2:** Apoptosis.

	control	debris	LIPUS+debris
Apoptosis rate	3.772 4.548	5.102 6.236	6.472 5.467
Mean ± SD	4.160 ± 0.549	5.669 ± 0.802	5.970 ± 0.711
