# Supplementary material for: Low-intensity pulsed ultrasound (LIPUS) prevents periprosthetic inflammatory loosening through FBXL2-TRAF6 ubiquitination pathway
Source: Sci Rep. 2017 Apr 5;7:45779. doi: 10.1038/srep45779 (PMC5381120; doi:10.1038/srep45779)
Supplement: Supplementary Information [file srep45779-s1.pdf]

Low-intensity pulsed ultrasound (LIPUS) prevents periprosthetic inflammatory loosening through FBXL2-TRAF6 ubiquitination pathway

Xiang Zhao[a,b,+] PhD, Gangsheng Zhao[c,+] MD, Zhongli Shi[a,b] PhD, Chenhe Zhou[a,b] PhD, Yunlin Chen[d] MD, Bin Hu PhD[a,b], and Shigui Yan[a,b,*] MD

[a]Department of orthopaedic surgery, the second affiliated hospital , School of Medicine, Zhejiang University, Hangzhou, China

[b]Institute of orthopaedic research, Zhejiang University, Hangzhou, China

[c]Department of Orthopaedic Surgery, Yiwu Central Hospital, the affiliated hospital of Wenzhou Medical College, Yiwu, China

[d]Department of Orthopaedic Surgery, Ningbo sixth hospital, China

+ These authors contributed equally to this work.

***Corresponding to**: Shigui Yan, zrjwsj@163.com

Supplementary table S1: The primer used in this study

Gene	Forward Primer	Reverse Primer
β-actin	5'- CCGAGAATGGGAAGCTTGTC-3'	5'- AAGCACCAACGAGAGGAGAA-3'
FBXL2	5'- CAGCCATCTTACGACACCAC-3'	5'- TTGCCAGAGAATCACCGTAG-3'
TRAF6	5'- AAACTGTGAGAACAGCTGCG-3'	5'- GCAGATGGGACACTCATACT-3'
ERK1	5'- GAAAGCATTACCTTGACCAG -3'	5'- CTTTGGAGTCAGCATTTGGG -3'
ERK2	5'- GGCATGGTTTGCTCTGCTTA -3'	5'- TCAATGGTTGGTGCCCGGAT -3'

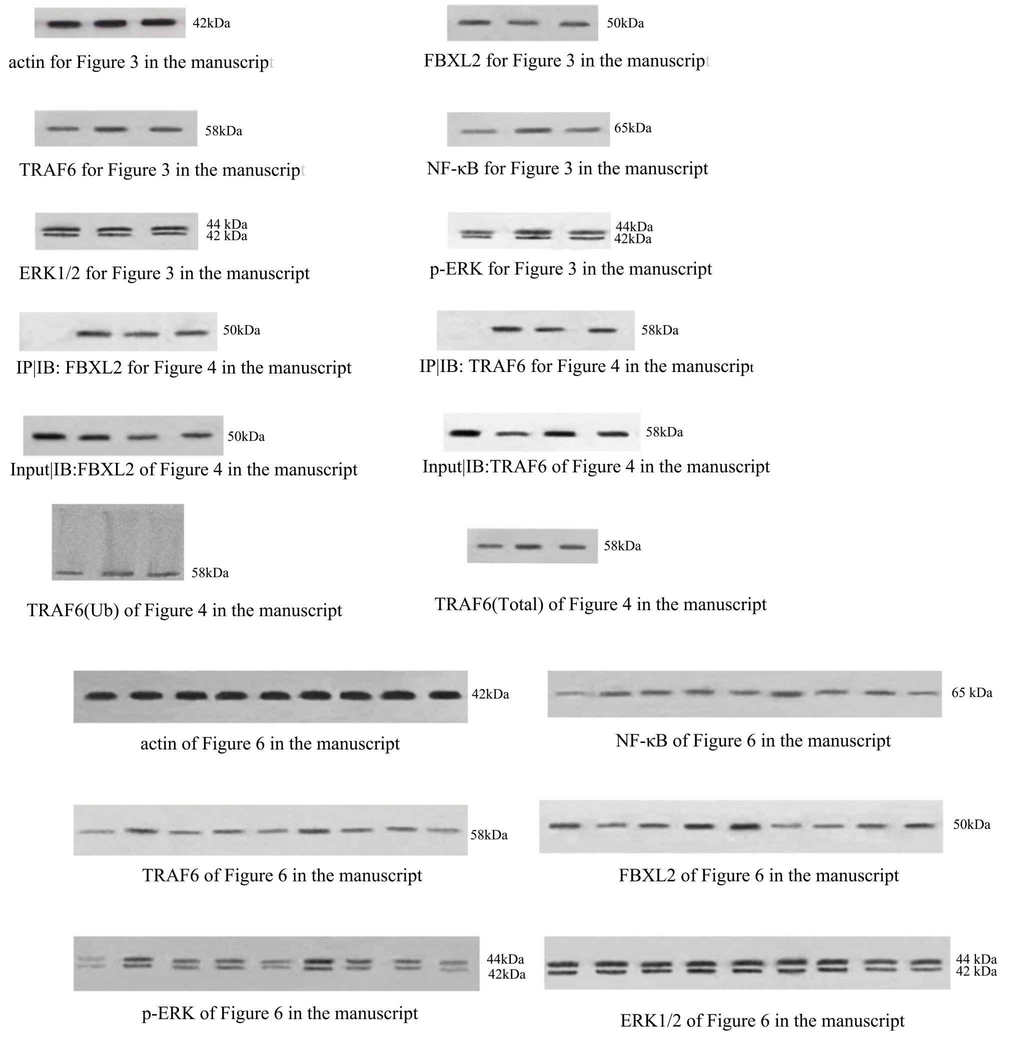

Supplementary Figure S1: the full-length blots used in the manuscript.
